# Structure and evolutionary history of a large family of NLR proteins in the zebrafish

**DOI:** 10.1098/rsob.160009

**Published:** 2016-04-27

**Authors:** Kerstin Howe, Philipp H. Schiffer, Julia Zielinski, Thomas Wiehe, Gavin K. Laird, John C. Marioni, Onuralp Soylemez, Fyodor Kondrashov, Maria Leptin

**Affiliations:** 1 Wellcome Trust Sanger Institute, Cambridge, UK; 2 Institut für Genetik, Universität zu Köln, Köln, Germany; 3 The European Molecular Biology Laboratory, Heidelberg, Germany; 4 The European Molecular Biology Laboratory, The European Bioinformatics Institute (EMBL-EBI), Wellcome Trust Genome Campus, Hinxton, Cambridgeshire, UK; 5 Bioinformatics and Genomics Programme, Centre for Genomic Regulation (CRG) 88 Dr. Aiguader, 08003 Barcelona, Spain; 6 Universitat Pompeu Fabra (UPF), 08003 Barcelona, Spain; 7 Institució Catalana de Recerca i Estudis Avançats (ICREA), 23 Pg. Lluís Companys, 08010 Barcelona, Spain

**Keywords:** NACHT, B30.2, SPRY, gene conversion, innate immune system, genome evolution

## Abstract

Multicellular eukaryotes have evolved a range of mechanisms for immune recognition. A widespread family involved in innate immunity are the NACHT-domain and leucine-rich-repeat-containing (NLR) proteins. Mammals have small numbers of NLR proteins, whereas in some species, mostly those without adaptive immune systems, NLRs have expanded into very large families. We describe a family of nearly 400 NLR proteins encoded in the zebrafish genome. The proteins share a defining overall structure, which arose in fishes after a fusion of the core NLR domains with a B30.2 domain, but can be subdivided into four groups based on their NACHT domains. Gene conversion acting differentially on the NACHT and B30.2 domains has shaped the family and created the groups. Evidence of positive selection in the B30.2 domain indicates that this domain rather than the leucine-rich repeats acts as the pathogen recognition module. In an unusual chromosomal organization, the majority of the genes are located on one chromosome arm, interspersed with other large multigene families, including a new family encoding zinc-finger proteins. The NLR-B30.2 proteins represent a new family with diversity in the specific recognition module that is present in fishes in spite of the parallel existence of an adaptive immune system.

## 1. Background

The need to adapt to new environments is a strong driving force for diversification during evolution. In particular, pathogens, with their immense diversity and their ability to subvert host defence mechanisms, force organisms to develop ways to recognize them and keep them in check. The diversity and adaptability of pathogen recognition systems rely on a range of genetic mechanisms, from somatic recombination, hypermutation and exon shuffling, to gene conversion and gene duplication to generate the necessary spectrum of molecules.

NACHT-domain- [1] and leucine-rich-repeat-containing (NLR) proteins (reviewed in [2,3]) can act as innate immune sensors for sterile and pathogen-associated stress signals in all multicellular organisms. Members of this protein family have also been called NOD-like receptors or nucleotide-binding domain and leucine-rich-repeat-containing proteins [3–6]. The protein families to which these names refer are largely but not completely overlapping. Thus, some members, such as Apaf1, do not contain LRRs, and others do not act as ‘receptors', even though they contain LRRs.

In vertebrates, eight conserved NLR proteins are shared across a wide range of species. These are the transcriptional regulators CIITA and NLRC5, the inflammasome and nodosome proteins NOD1, NOD2, NOD3/NlrC3, Nod9/Nlrx1, and the as yet functionally uncharacterized NachtP1 or NWD1 [7,8]. An eighth member, the sensor for apoptotic signals, APAF1, is often included in the family, although the nucleotide-binding domain is not strictly a NACHT domain, and it has WD40 repeats instead of LRRs. Other NLR proteins, which must have evolved independently of the conserved set, are shared by only a few species, or are unique to a species. Non-vertebrates lack an adaptive immune system and can therefore be expected to benefit from an expansion of innate immune sensors. Indeed, very large families of NLR-encoding genes have been described in sea urchins and corals [9]. Surprisingly, an extreme example of species-specific expansion can be found in zebrafish despite the presence of an adaptive immune system [7]. Such species-specific gene family expansions suggest adaptive genome evolution in response to specific environments, most probably different pathogens [10].

**Figure 1.**
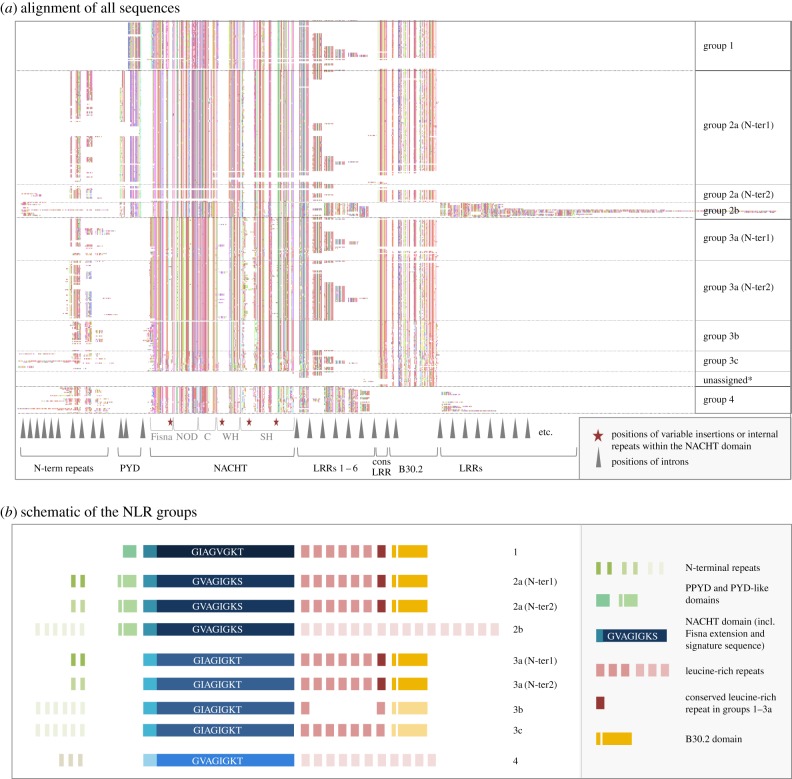
Structure of the fish-specific NLR-B30.2 protein family members. (*a*) Overview of an alignment of the entire set of 368 predicted NLR-B30.2 proteins in the zebrafish, based on a Clustal–Omega alignment. The original alignment (electronic supplementary material) was edited by hand to improve the alignments of the N-terminal repeats and the LRRs. The colour coding is a random assignment of colours to amino acids created in Jalview. Gaps were manually introduced in the alignment at the positions of introns (marked by a grey arrowhead below the alignment), except between the C-terminal extensively duplicated LRRs in group 2b and the extensively duplicated N-terminal repeats in groups 2b and 3b. A gap was inserted between the LRRs 6 and 7 of groups 2b and 3b to allow the conserved C-terminal LRR and the B30.2 domains of groups 1, 2a and 3a to be positioned immediately after the 1–6 LRRs in these groups. Further large gaps are created, because some positions are prone to variable and often long insertions or internal duplications (marked by red stars below the alignment). Some domains are present only in subsets of the genes; these include death-fold domains in group 1 and 2a, a B30.2 domain and various N-terminal peptide repeats. Group 2 consists of two subgroups, the large and very homogeneous group 2a and the smaller group 2b, which is restricted to a cluster on chromosome 22. Group 3 has several subgroups that differ in their N-terminal peptides, their LRRs, or their B30.2 domains. *unassigned: the set of incomplete genes that lacked a NACHT domain could not be assigned to a group. (*b*) A schematic representation of the protein domains in each group, on the same scale as in the alignment above. Each box represents an exon. A defining motif for each group is the sequence of the Walker A motif, for which the consensus over the whole set is G[IV]AG[IV]AGK[TS]. All four groups share the Fisna domain, the NACHT domain and the LRRs. Please refer to the alignment file (electronic supplementary material) for the many details not captured in this simplified figure.

The zebrafish has become a widely used model system for the study of disease and immunity [11,12], and a good understanding of its immune repertoire is necessary for the interpretation of experimental results, for example in genetic screens or in drug screens. In a previous study, we discovered more than 200 NLR-protein encoding genes [7]. The initial description and subsequent analyses [13,14] have led to the following conclusions: the zebrafish-specific NLRs have a well-conserved NACHT domain (PF05729), with an approximately 70 amino acid upstream extension, the Fisna domain (PF14484). This domain characterizes this class of NLR proteins and is found in all sequenced teleost fish genomes, but not outside the fishes [7]. The NLR proteins can be divided into four groups, each defined by sequence similarity in the NACHT and Fisna domains; these groups also differ in their N-terminal motifs. Groups 1 and 2 have death-fold domains; groups 2–4 contain repeats of a peptide motif that is only found in this type of NLR protein (figure 1).

In the initial description, all of the novel NLR proteins ended with the leucine-rich repeats (LRRs), but others found that several had an additional C-terminal domain, an SPRY/B30.2 domain (PF00622), which also occurs in another multigene family implicated in innate immunity, the fintrims [14].

Large expansions of immune gene families, as seen in the NLR-protein encoding genes, could have occurred to allow high expression levels, or to allow adaptation to a diverse pathogen fauna. If there was pressure to respond to different pathogens, one would expect to see the signs of diversification between the paralogues, and perhaps evidence for selection on domains in the diversified proteins. The difference between paralogous subfamilies may be maintained by selection or be the consequence of neutral evolution.

The initial identification of the genes and subsequent analyses suffered from the limitations of the then available Zv6 assembly and gene annotations (published in 2006). In particular, there was a limited amount of data available for long-range assembly arrangements and a lack of supporting evidence for gene models, such as well-annotated homologues from other species. In addition, the very high similarity of the NLR genes, as well as their clustered arrangement in the genome, further complicated the assembly. As a result, many genomic regions were collapsed, and many of the gene models were incomplete and their genomic location incorrect. These shortcomings made it impossible to address questions about the composition and the evolution of this family.

We have re-analysed this gene family to identify all members and improve the genome assembly in the regions of interest. We have manually annotated and refined the structure of more than 400 genes and provide a full description of the protein domain arrangements, genomic distribution and evolutionary history. This allowed us to elucidate the mechanism underpinning diversity in signal recognition while maintaining protein similarity. In particular, we explored whether the accumulation of neutral substitutions allowed sequences to escape from intrasubfamily gene conversion.

## 2. Results

### 2.1. Identification of all NLR genes in the zebrafish genome

To identify the entire set of fish-specific NLR encoding genes in the zebrafish genome, we used various approaches to collect lists of candidate genes based on the Zv9 assembly (GCA_000002035.2; for details, see Methods and electronic supplementary material, Methods). We identified genomic regions containing domain motifs via hmmsearch (hmmer.janelia.org/search/hmmsearch), electronic PCR [15], TBLASTN searches and by mining the existing annotations for keywords. This collection was purged of gene models belonging to other known families, e.g. fintrims. We identified all overlapping Ensembl and VEGA gene models for the remaining regions of interest. The VEGA gene models were refined and extended through manual annotation and both gene sets merged, resulting in 421 NLR gene models.

Beyond the eight conserved NLR genes, and nine other NLR genes (table 1) that had a different structure from those described previously and below, the zebrafish genome contains 405 genes (368 protein-coding and 37 pseudo-genes) encoding NLR proteins that are members of the family we had previously called ‘novel fish NLR proteins’ [7]. Henceforth, we will refer to these as NLR-B30.2 proteins (see below, also electronic supplementary material, Figshare: http://dx.doi.org/10.6084/m9.figshare.1473092). The genome assembly components carrying these gene models were checked for correct placement and relocated if necessary. The resulting corrections were incorporated into the GRCz10 assembly (GCA_000002035.3).

**Table 1.** All NLR proteins in the zebrafish genome.

Zf identifier	protein name
1. NLRs with orthologues in mammals	
ENSDARP00000052748	nod1
ENSDARP00000124380	nod2
ENSDARP00000102939	nlrc3/nod3
ENSDARP00000101928	nlrx1
ENSDARP00000099546	NWD1/NACHTP1
ENSDARP00000105957	CIITA
ENSDARP00000105810	Apaf1
ENSDARP00000126444	NLRC5
2. Other NLRs	
ENSDARP00000118135	NLRP6 (4 of 5)
ENSDARP00000105086	NLRP6 (1 of 5)
ENSDARP00000107209	NLRP6 (3 of 5) nlrb5
ENSDARP00000126513	NLRP6 (2 of 5)
ENSDARP00000104483	NLRP6 (5 of 5)
OTTDARP00000028005	—
ENSDARG00000088041/R4GEV1_DANRE	NLRC3-like
ENSDARG00000087736	—
3. Fish-specific NLR multigene family,	
see electronic supplementary material	

### 2.2. Domain structure of the NLR family members

The original set of 205 genes described in Stein *et al*. [7] was divided into four groups based on sequence similarity in the Fisna and NACHT domains. The extended and updated gene set confirmed this classification and revealed further aspects of the structure of the family. A defining motif for each group is the sequence of the Walker A motif, but the groups also differ in the composition of their domains, as summarized in figure 1.

**Figure 2.**
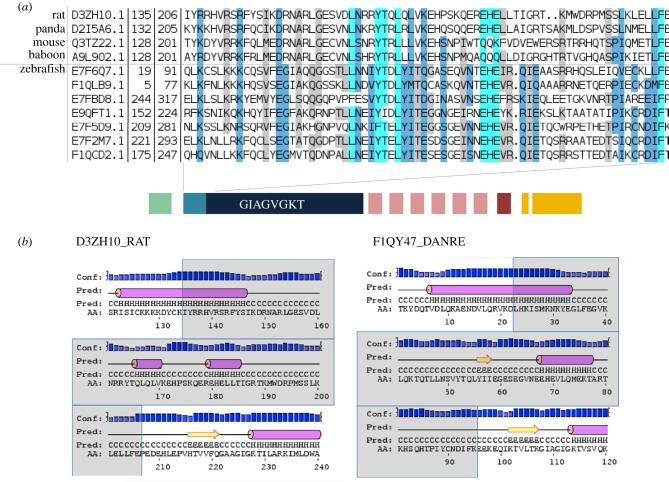
The Fisna domain and its homologues in mammalian proteins. (*a*) Alignment of sequences identified in mammalian genomes using an HMM search with PF14484 and examples of zebrafish Fisna domains. (*b*) Secondary structure predictions for selected examples.

The Fisna domain was previously found only in fish NLR proteins. We used our new collection of Fisna sequences to build a hidden Markov model defining a Pfam family, deposited as PF14484, and searched for homology with mammalian proteins. This revealed alignments with high significance to mammalian members of the NLR family (NLRP3 and NLRP12), with the matching sequence located immediately upstream of the NACHT domain, i.e. in the homologous position to the Fisna domain, suggesting it should be considered a subdomain of the NACHT domain. Secondary structure predictions based on two representatives from zebrafish and the rat using the PSIPRED workbench suggest that the zebrafish Fisna domain may take on the same conformation as the corresponding mammalian proteins (figure 2). Together, these findings suggest that the Fisna subdomain was present in the common ancestor of mammalian and fish NLR proteins. We did not find Fisna domains in non-vertebrate genomes.

The N-terminal extensions of groups 2, 3 and 4 contain several repeats of an approximately 30 amino acid peptide motif, of which there are two main types. Surprisingly, each of the main types of N-terminal repeat can associate with either group 2 or group 3 NACHT domains, indicating extensive exon-shuffling between family members (figure 1: group 2a (N-ter1) and group 2a (N-ter2)). We also see no correlation between the type of N-terminal repeat and other parts of the protein, such as the LRRs or the B30.2 domains.

The LRRs (Pfam Clan: CL0022) in groups 1, 2a and 3 are of two types: the last LRR, immediately upstream of the B30.2 domain, occurs in each gene exactly once, and barely differs between genes (electronic supplementary material, figure S2). The other type varies in number between 0 and 6, and has more divergent sequence. Thus, similar to the situation in the lamprey variable lymphocyte receptors [16], the C-terminal LRR seems to be fixed, whereas the others vary more and are duplicated to varying degrees. Groups 2b and 4 do not show this arrangement, but they have yet another type of LRR, which can occur more than 20 times.

A B30.2 domain has so far been reported in some but not all fish NLR proteins [13,14]. We find that the B30.2 domain is restricted to groups 1, 2a and 3, and is lacking in all members of groups 2b and 4 (figure 1 and electronic supplementary material, figure S3). In view of the extreme similarity between the N-terminal parts (NACHT and death-fold domain) of groups 2a and 2b, and the overall conservation of the gene structure throughout the whole family, it seems most likely that the B30.2 domain was present in the common ancestor of the family but lost by groups 2b and 4, rather than independently gained by the other groups. We therefore refer to the entire family as the NLR-B30.2 protein family.

All genes in this family have the same exon–intron structure (figure 1). The largest exon contains the NACHT domain with the N-terminal Fisna extension and the winged helical and superhelical domains, as is also the case in NLRC3, for example. All other domains (N-terminal peptides, LRRs, B30.2 domain) are each encoded on single exons.

### 2.3. Divergence of NACHT and B30.2 domains

The sequence alignments show little divergence of the NACHT domains within each of the groups 1, 2a and 3a, but strong divergence between different groups (figure 1 and electronic supplementary material, figure S1). In contrast, there is no recognizable group-specific sequence pattern in the B30.2 domain. This observation is supported by independent phylogenetic analyses of the domains that yield different tree topologies (electronic supplementary material). The NACHT domains cluster into the same monophyletic groups that were obtained with the entire sequence and that formed the basis for the definition of the groups. However, the tree for the B30.2 domain does not recapitulate this pattern. The only B30.2 domains that cluster together are those from group 3b. Some of the B30.2 domains from group 1 are found on one branch, but others are more related to those of groups 2 and 3. No group-specific clustering can be seen for the B30.2 domains of groups 2a and 3a. The discrepancy between the trees suggests different evolutionary trajectories for the B30.2 and NACHT domains. Thus, on the one hand, proteins with different NACHT domains share similar B30.2 domains, and on the other, proteins with nearly identical NACHT domains and N-terminal motifs, such as those in groups 2a and 2b, have different C-termini.

Both the shuffling of N-termini and the unequal divergence of the NACHT and B30.2 domains suggest a complex evolutionary history of the gene family. To analyse the divergence, we calculated the rates of non-synonymous and synonymous substitutions in the NACHT and B30.2 domains (dN, dS and dN/dS values). We studied only those groups that show high intragroup conservation of the NACHT domains (groups 1, 2a, 3a and 3b). We considered groups 3a and 3b separately, because inspection of the protein alignment suggested that although they are assigned to the same group by virtue of their NACHT domain, their B30.2 domains had diverged. We omitted group 3c, as its NACHT domains are more divergent, and may represent further groupings. Median values are in table 2, and all data are displayed in the electronic supplementary material, figure S4.

**Table 2.** Median dN (*a*), dS (*b*) and dN/dS (*c*) values calculated from all pairwise comparisons in the exons coding for the NACHT domain and the B30.2 domain. For dS, values below 0.2 are in italics and for dN/dS, values higher than 1 are in italics.

	Fisna–NACHT exon				B30.2 exon			
	group 1	group 2a	group 3a	group 3b	group 1	group 2a	group 3a	group 3b
(*a*) dN								
group 1	0.058	0.44	0.497	0.482	0.097	0.094	0.1	0.398
group 2a		0.029	0.411	0.398		0.09	0.096	0.398
group 3a			0.038	0.11			0.107	0.4
group 3b				0.042				0.226
(*b*) dS								
group 1	*0.116*	1.799	1.684	1.660	*0.067*	*0.061*	*0.066*	1.119
group 2a		*0.043*	1.525	1.491		*0.053*	*0.058*	1.091
group 3a			*0.038*	0.216		* *	*0.065*	1.069
group 3b				*0.094*				0.445
(*c*) dN/dS								
group 1	0.52	0.245	0.292	0.291	*1.444*	*1.513*	*1.569*	0.352
group 2a		0.639	0.267	0.267	* *	*1.589*	*1.667*	0.361
group 3a			0.942	0.506	* *	* *	*1.707*	0.371
group 3b				0.677				0.497

The median rate of synonymous sequence substitutions in the NACHT domains was very low when comparing members within a group (0.01–0.05). The values for comparisons between groups were 20- to 100-fold higher. Only the group 3a to 3b comparison resulted in a low value, confirming their classification by NACHT domain as a single group.

The B30.2 domains showed a different pattern. Similar to the NACHT domain, the median dS values for comparisons within each group ranged between 0.02 and 0.04 for groups 1, 2a and 3a. However, for the B30.2 domain, the values for the between-group comparisons were low: they were minimally or not at all higher than for within-group comparisons. The B30.2 domain sequences of the members of group 3c were more divergent, both from each other and from those in groups 1, 2a and 3a (table 2 and electronic supplementary material, figure S4). The patterns of synonymous divergence between groups were clearly different for NACHT and B30.2 domains and confirmed the different behaviour of the two domains suggested in the alignment of the protein sequences. The observed patterns are most easily explained by gene conversion (see Discussion). Interrogating the dN/dS values for these comparisons reveals a second evolutionary mechanism acting in the B30.2 domain. We find high median dN/dS values, consistent with positive selection acting on the B30.2 domain (electronic supplementary material, figure S4). Thus, we confirm and extend a previous conclusion for the B30.2 domain in the fintrim proteins [14], namely that positive selection probably creates variation for pathogen recognition in this domain. We discuss below how the combination of positive selection and gene conversion may have created variation within the B30.2 domain throughout the entire family.

### 2.4. Origin of the NLR-B30.2 gene groups

The degree of apparent gene conversion within the NLR-B30.2 gene family makes it difficult to judge when the groups arose or expanded during fish evolution. Moreover, the high divergence between the four groups suggests the split may be old. To explore whether the groups arose in zebrafish or in an ancestral species, we compared the NLR-B30.2 genes in zebrafish with those in the closest relative for which a whole genome sequence exists, the carp, as well as NLR-B30.2 genes in other vertebrate genomes (see Material and methods).

**Figure 3.**
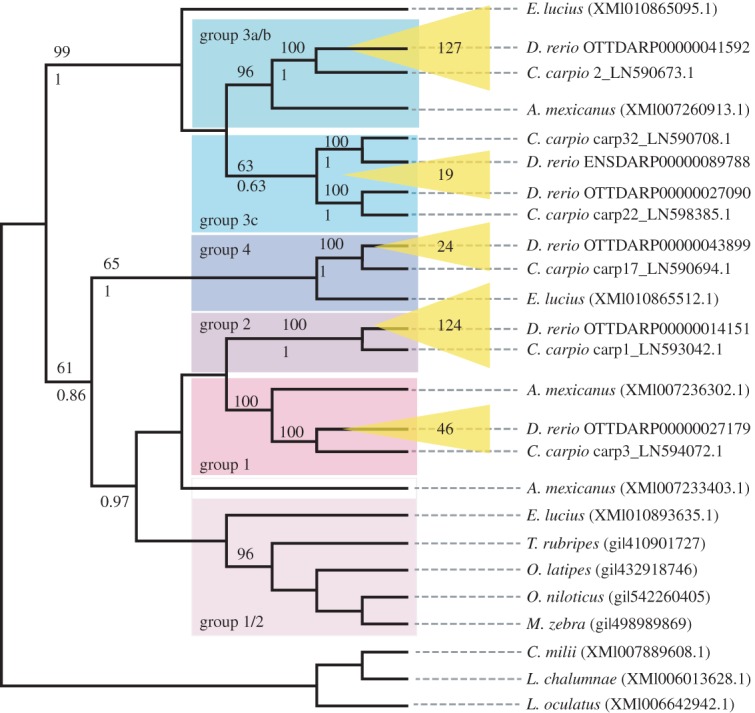
Relationships between NLR-B30.2 genes in different fishes. Phylogenetic tree resulting from a recursive phylogenetic analysis. Expansions of groups in zebrafish are indicated in yellow with numbers of genes per group displayed. Bootstrap values are given above each branch where higher than 50% and pp values (from MrBayes) below each branch, where they are higher than 50% and the topology is congruent.

A tree resulting from a recursive phylogenetic analysis indicates that the split into groups occurred before the zebrafish–carp divergence (figure 3). Groups 1, 2, 3a/b and 4 have orthologous relationships between carp and zebrafish: for example, group 2 from zebrafish is most closely related to a group of genes in the carp that is distinct both from other carp NLR-B30.2 genes and from the other zebrafish genes (see also a tree containing all available zebrafish and carp genes in the electronic supplementary material, figure S5). Not unexpectedly, group 3c, which has a more heterogeneous set of sequences in the zebrafish, shows a more complex evolutionary history. It falls into two groups, both of which have an orthologous group in the carp. *Astyanax mexicanus* (Mexican tetra) and *Esox lucius* (Northern pike) each have groups of genes that cluster with groups of the zebrafish genes, rather than with each other, but not every group is represented in each of the species. Nevertheless, this suggests that the split is even older than the carp–zebrafish split, having perhaps occurred in basal teleosts. Finally, sequences from *Lepisosteus oculatus* (spotted gar)*, Latimeria chalumnae* (West Indian Ocean coelacanth) and *Callorhinchus milii* (Australian ghostshark) do not fall into these groups, suggesting that the split into groups must have occurred soon after the emergence of the teleosts, in the branch of the Clupeocephala.

In summary, the groups did not diverge independently from duplicated ancestral genes in each species, but already existed in a common precursor. By contrast, within a species, the majority of genes arose by independent amplification of a founding family member.

### 2.5. Co-occurrence of the NACHT and B30.2 domains

As first reported by van der Aa *et al*. [14], the NLR-B30.2 domain fusion is found in all teleost fish. Our collection of NLR-B30.2 genes showed that this domain fusion arose prior to the common ancestor of teleosts, as it was also present in the spotted gar (see for example ENSLOCG00000000593), for which the genome sequence was not previously available. The NLR-B30.2 proteins predicted in the spotted gar also contain an N-terminal Fisna extension, though only distantly related to those in the teleosts, indicating that the ancestral gene included this extension. This is consistent with the fact that the N-terminal extension of the mammalian NLRP3 proteins has recognizable similarity to the Fisna domain, as described above.

We do not find evidence of the NLR-B30.2 fusion in any of the tetrapod genomes, nor in the coelacanth (*L. chalumnae*) or ghostshark (*C. milii*) genomes. These results indicate that the fusion occurred at least in the common ancestor of the Neopterygii subclass of the ray-finned fish, prior to the third whole genome duplication in the teleost lineage. Genome sequence data from fishes of other subclasses, such as sturgeon, paddlefish or bichir clades, would provide further information on the point of emergence of the NLR-B30.2 fusion, but are currently not available.

### 2.6. Expansion of the NLR-B30.2 family in fish

The phylogeny of the NLR-B30.2 gene family also provides information on the timing of the lineage expansion of the NLR-B30.2 genes observed in the zebrafish. The *Cyprinus carpio* (carp) genome contains a similarly large family of NLR-B30.2 proteins. However, because of the polyploidy of the species, it is impossible to know whether some of the nearly identical sequences constitute paralogues or different alleles. This will perhaps be resolved in future improved assemblies of the genome. The Mexican tetra (*A. mexicanus*), a direct outgroup to the zebrafish–carp clade, features the second largest NLR-B30.2 gene family with approximately 100 members, showing that the lineage expansion began prior to the zebrafish–carp split. Other fish species, including the spotted gar, have fewer than 10 NLR-B30.2 genes, whereas the Northern pike (*E. lucius*), from the euteleost clade, has approximately 50. Thus, the initial gene expansion either occurred in the basal branches of Teleostei with a subsequent loss in some lineages, or independently in several lineages. Independent expansions and losses are a likely scenario, given the expansions of NLR genes in many other species, such as sponges and sea urchins [17,18]. The results on fish show that expansions of the NLR-B30.2 family genes began as soon as the NLR-B30.2 fusion occurred, with different dynamics in different lineages.

### 2.7. Evolutionary age of the NLR-B30.2 family relative to conserved NLRs

**Figure 4.**
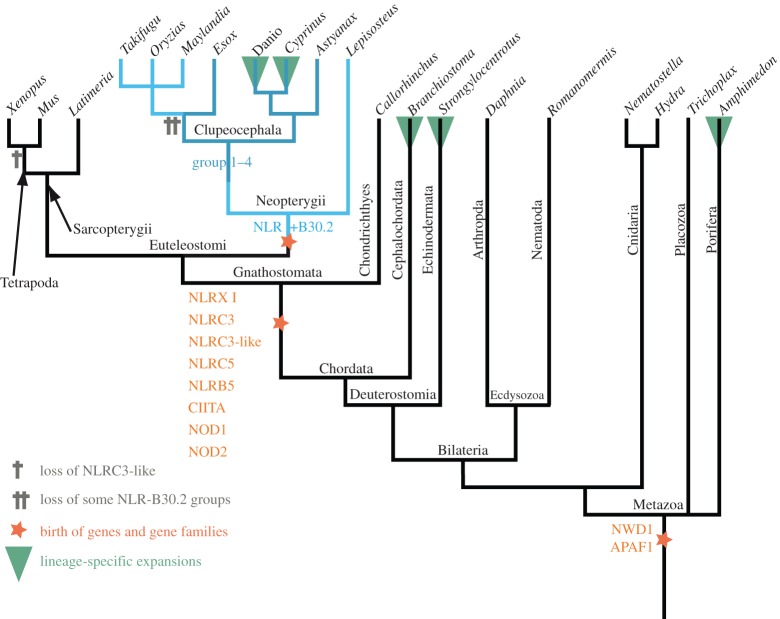
Evolutionary history of NLR genes. A reduced dendrogram of metazoa based on the NCBI taxonomy database displaying key events in the evolution of NLR genes as described in the main text. See electronic supplementary material for phylograms and figure S6.

We compared the origin of the NLR-B30.2 gene family with the evolutionary history of other NLR genes (figure 4). There are many species-specific expansions of NLRs, such as the Nalp proteins in mouse and human and the NLR-B30.2 genes in fish, as well as independent expansions in amphioxus, sea urchin and sponge. However, there also exist the eight NLR proteins that are conserved in all vertebrates and show orthologous relationships. We collected the available orthologues of the conserved NLRs from key metazoan species and created an alignment that also included all NLR genes from fish that did not belong to the NLR-B30.2 group (listed above and in Methods).

**Figure 5.**
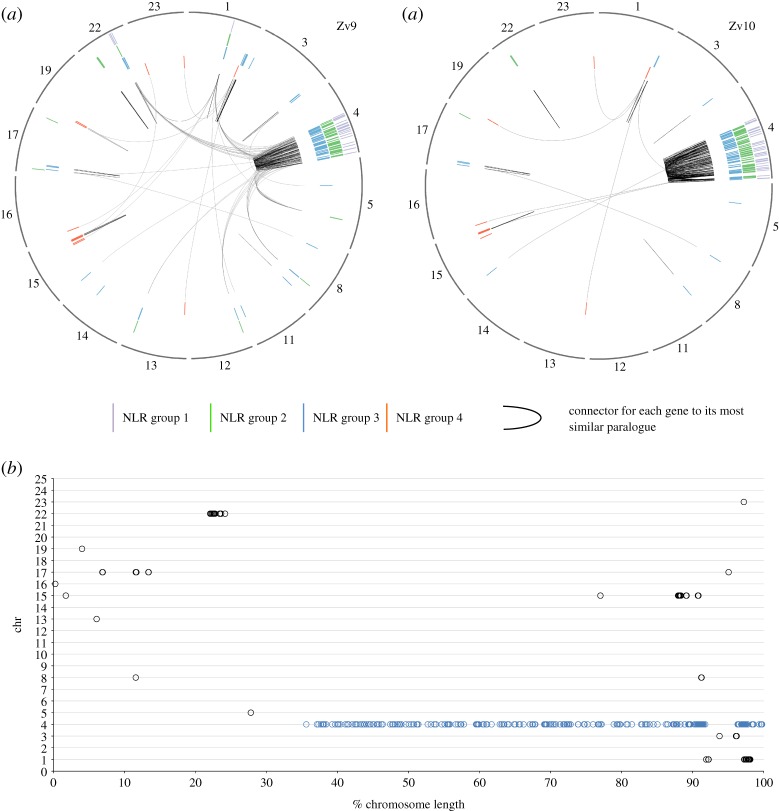
Location of the NLR genes in the genome in assemblies Zv9 and GRCz10. (*a*) The chromosomes containing NLR-B30.2 genes are shown in the outer circle (note that corrections of the genome between Zv9 and the GRCz10 have changed the lengths of some of the chromosomes. The genes were annotated on Zv9 and lifted over to the GRCz10 path where possible as the GRCz10 gene set did not become available until May 2015. The members of the four groups of NLR genes are shown as radial lines within the circles with group 1 in the outermost and group 4 in the inner ring. Each gene is connected by a black line to its most closely related paralogue, based on the number of amino acid substitutions per site calculated in MEGA5 (Poisson correction model). Most genes are most closely related to a near neighbour, resulting in a line reaching towards the centre and returning to nearly its origin (for example, the group 2 genes on chromosome 22). The changes in the assembly have led to many genes that were closely related but resided on different chromosomes in Zv9 being located in closer proximity in GRCz10. (*b*) Normalized location of NLR-B30.2 genes on chromosomes. Each chromosome is shown as a horizontal line of 100% length, and the NLR genes are plotted at their relative positions along the chromosome. Apart from the genes on chromosome 4 (marked in blue), all other genes are found within the first or last quarter of the chromosome.

We found that two of the conserved vertebrate NLR genes appear to be shared by all animals (figure 5 and electronic supplementary material, figure S5). The genes for NWD1 (first described in zebrafish as NACHT-P1) and Apaf1 must have been present in the last common ancestor of bilaterians and non-bilaterians, as they are found in sponges, cnidarians and all bilaterians analysed. We could not find any candidates in comb jellies (ctenophores). The other five conserved NLR proteins—Nod1, Nod2, NLR3C, CIITA and NLRX1—arose later in evolution, at the base of the gnathostomes. An additional gene, NLR3c-like, was present at this point, but appears to have been lost in the tetrapod lineage (also see electronic supplementary material, figure S6).

In summary, all of the conserved vertebrate NLRs are older than the NLR-B30.2 family. They are never duplicated and certainly not expanded to higher gene numbers in any species.

### 2.8. Genomic location of the NLR-B30.2 genes

The first survey of NLR genes on the zebrafish genome assembly Zv6 suggested that they were located on 22 different chromosomes, with some enrichment on chromosome 4 (50 genes) and chromosome 14 (47 genes) [7]. Since this analysis, the assembly of the zebrafish genome has been significantly improved, and the current Zv9 NLR gene set shows a more restricted distribution (figure 5), with 159 (44%) of the genes located on the long arm of chromosome 4. The remaining genes are distributed between 12 other chromosomes (153 genes) and unplaced scaffolds (56 genes).

Additional sequencing and data gathering by the Genome Reference Consortium since the release of Zv9 led to the rearrangement of multiple assembly components, including relocation of sequence to different chromosomes. These placements are based on manual curation by the Genome Reference Consortium, supported by genetic mapping data, clone end sequence placements and optical mapping data [19]. As part of the re-annotation of the NLR-B30.2 gene set, more than 50 locations of assembly components were queried and 12 were reassigned to new chromosomal positions. The latest assembly, GRCz10, reveals that the genomic location of the genes now reflects the domain-based classification. The majority of the genes are clustered on the long arm of chromosome 4, where 75% of the NLR-B30.2 genes, including all group 1 and group 2a genes, now reside (figure 5). Group 2b genes are now found exclusively in a cluster on chromosome 22, which suggests that they arose via local duplications of a single precursor gene that had lost its B30.2 domain. Similarly, group 3a genes are clustered together on chromosome 4, with group 3b and 3c genes arranged on chromosomes 1 and 17, respectively. Group 4 genes are found mostly on chromosome 15, some on chromosome 1, but are notably excluded from chromosome 4. Both group 2 and group 3 have a few individual genes dispersed over other chromosomes; careful inspection of the evidence on which this allocation is based revealed no indications that it is incorrect. Some of the group 3 members on other chromosomes are more divergent from the consensus for this group, suggesting they may indeed have separated from the group early. Within chromosome 4, no clear pattern can be detected in the distribution of the genes. We are, however, aware of possible shortcomings in the assembly of the long arm of chromosome 4; the highly repetitive nature of the sequence makes it difficult to exclude with absolute certainty shuffling of gene locations. In addition to containing multiple copies of 5S ribosomal DNA [20], 53% of all snRNAs and the majority of the NLR-B30.2 genes, chromosome 4 also contains multiple copies of genes encoding a particular type of Zn-finger protein, which we discuss below.

Finally, another striking feature of the genes' genomic location is that they tend to accumulate near the ends of the chromosomes (figure 5*b*). With the exception of the cluster on chromosome 22, and two single genes on chromosomes 5 and 15, all other genes (81% of the NLR genes outside chromosome 4) are located within 15% of chromosome ends. On chromosome 4, we found 26% of the genes within 15% of the end.

### 2.9. Distribution of fintrim and multiple Zn-finger encoding genes

We noted that the NLR-B30.2 genes on chromosome 4 were often interspersed with genes encoding multiple tandem Zn-finger proteins. In some cases, older gene models had joined B30.2 domains with Zn-fingers. However, our manual analyses showed that the B30.2-encoding exons in these automatically created gene models belonged to neighbouring NLR genes, rather than the more distant Zn-finger encoding exons. A possible explanation for the erroneous annotation is that the predictions created apparent *fintrim* (*ftr*) genes. Fintrim proteins, members of the larger tripartite motif (TRIM) protein family, are composed of multiple Zn-fingers combined with a B30.2 domain and are assumed to act as sensors for immune stimuli [21]. We therefore analysed the distribution of the NLR-B30.2 genes relative to the location of fintrims and multiple Zn-finger encoding genes.

**Figure 6.**
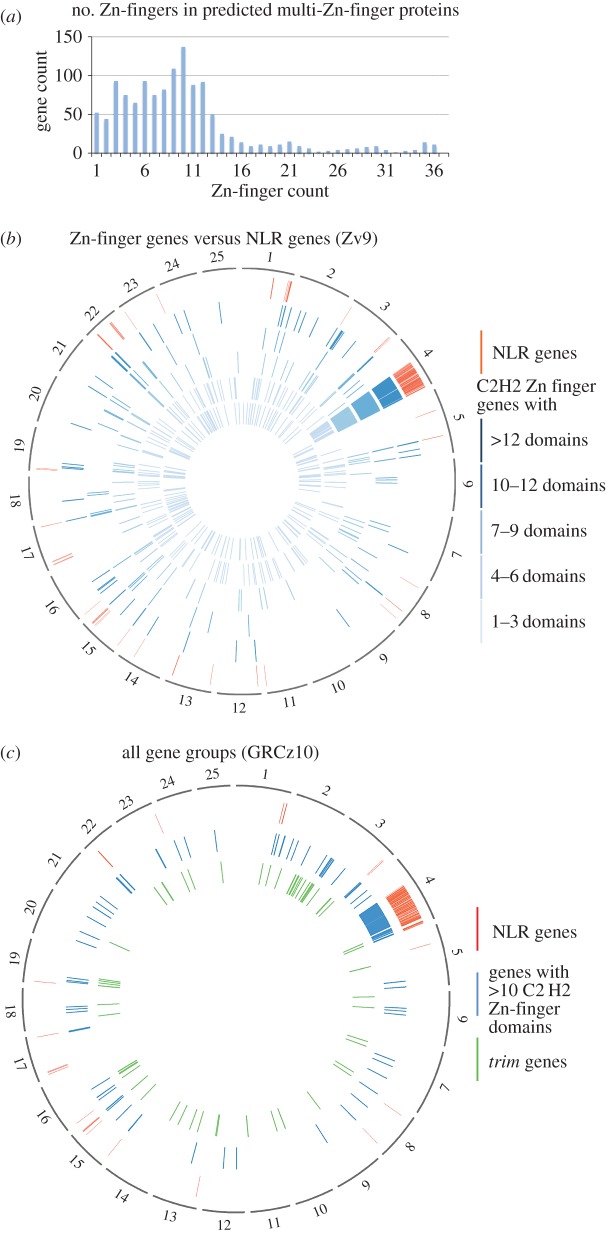
Genomic positions of genes for multi-Zn-finger proteins, fintrims and NLR-B30.2 proteins. (*a*) Frequency of genes encoding proteins with the indicated number of Zn-finger domains. Total number of Zn-finger encoding gene predictions: 1102. (*b*) The genomic locations of Zn-finger encoding genes is plotted on a circos diagram representing the Zv9 assembly for five subgroups defined by the number of Zn-finger domains. Inner circles (light to dark blue): 1–3, 4–5, 6–7, 8–9, 10–12 and more than 12 domains; outer circle (red): NLR genes. (*c*) Spatial distribution of NLR genes, *trim* genes and genes with at least 10 Zn-finger domains in the GRCz10 assembly (for gene liftover, see figure 5).

We established a list of *ftr*-related genes found in the zebrafish genome. This included 61 *trim* genes, 40 *ftr* genes and 18 genes of the related ‘*bloodthirsty*’ (*btr*) group (electronic supplementary material). The B30.2 domains from the NLR-B30.2 genes are more closely related to each other than to those of the *trim* families, and we found no close association in the genome between the *fintrim* and the NLR-B30.2 genes (figure 6*b*).

However, as noted above, genes encoding multiple Zn-fingers consisting exclusively of tandem repeats of Zn-fingers of the classical C2H2 type (IPR007087) were interspersed among the NLR-B30.2 genes. Unlike the *trim* genes, which contain C3HC4 (RING) and Znf-B-box domains (IPR001841 and IPR000315, respectively), the genes on chromosome 4 encoded yet uncharacterized proteins. We found 1259 gene models encoding Zn-fingers of this type, with the number of repeats per gene ranging from 1 to 36.

The encoded proteins with small numbers of Zn-fingers included many known proteins, including the Sna and Opa transcription factor families. These genes were dispersed broadly throughout the genome and largely excluded from chromosome 4 (figure 6). By contrast, genes encoding those proteins with larger numbers of Zn-finger domains are progressively clustered in restricted regions of the genome. For example, the majority (66%) of genes with more than 10 C2H2 domains are found on the right arm of chromosome 4, where they are interspersed in an irregular pattern among the NLR-B30.2 genes. Outside chromosome 4, some multi-Zn-finger genes co-locate with subsets of the *trim* genes, for example on chromosomes 3, 16 and 19, whereas others are located in regions where neither NLR-B30.2 genes nor *trim* genes are found. Similar to the NLR-B30.2 genes, multi-Zn-finger genes outside of chromosome 4 tend to be close to chromosomal ends (62% of genes within 15%). On chromosome 4, however, only 8% of the multi-Zn-finger genes are found within 15% of the chromosome ends.

In summary, the local duplications that may have led to the expansion of the NLR-B30.2 genes on chromosome 4 may also have duplicated the multi-Zn-finger genes, which have subsequently been transposed to other chromosomes.

## 3. Discussion

### 3.1. Phylogeny of vertebrate NLR proteins

The family of NLR-B30.2 genes has been shaped by different genomic and genetic mechanisms throughout evolution. These include repeated gene amplifications, shuffling of exons and gene fusions, gene conversion and positive selection for diversity. The oldest NLR genes appear to be those encoding the ancestors of two conserved NLRs, Apaf1 and NWD1, which we find in all animal lineages. These proteins have not been reported to have immune functions. Apaf1, originally discovered as CED-4 in *Caenorhabditis elegans*, is an ancient regulator of apoptosis. The so far only function reported for NWD1, first identified in the zebrafish genome as NACHTP1 [7], is its involvement in androgen signalling in the context of prostate cancer [22]. It will be interesting to learn whether this is a special case of a more general immune function yet to be discovered, or whether, like Apaf1, this old gene does not have immune functions. The other conserved genes first appear at the base of the jawed vertebrates, and all have roles in immunity or inflammation, whether as transcription factors or as inflammasome components.

NLR genes have duplicated and often undergone extensive species-specific expansions throughout evolution. This is the case, for example, for the members of the Nalp/NLRP family in the mouse and the NLR-B30.2 family we discuss here. The largest of the known early expansions were in the sponge *Amphimedon queenslandica*, the sea urchin *Strongylocentrotus purpuratus* and the lancelet *Branchiostoma floridae*, with 120, 92 and 118 genes, respectively [17,23]. As more genomes are sequenced, it is likely that additional NLR expansions will be discovered. In vertebrates, the largest expansions are those of the NLR-B30.2 family, although we also find other NLR gene families, for example in the Australian ghostshark *C. milii* (electronic supplementary material, figure S7).

The expansion of the families argues in favour of their involvement in immunity or broader stress reactions, as seen in numerous other examples of expanded gene families. Expansions can increase the amount of gene product, for example to adapt to stressful environmental conditions [24,25], as in the cold adaptation in several gene families expressed in Antarctic icefish [26]. Expansions can also allow the creation of the variety of sequences that are needed for immune recognition, as in the case of antibodies and T-cell receptors, or the more recent example of the VLR genes in lampreys and hagfish [27].

The likely scenario for the origin of the current NLR-B30.2 gene family in the zebrafish is their initial creation through the fusion of the NLR and B30.2 components early in the fish lineage and subsequent duplications, similar to many other NLR genes in other lineages [17,28]. Unfortunately, the available data are not sufficient to trace these earliest duplications. In particular, the extensive expansion of the NLR-B30.2 genes in fishes is remarkably similar to the evolution of the *trim* genes in vertebrates, which also contain a B30.2 domain that has been extensively diversified [29].

The paralogues that were present in the common ancestor of the teleost lineage then diversified into groups in the Clupeocephala superorder. Whether the common ancestor of the Clupeocephala had four genes (or a similarly small number) or whether each of the four genes had already begun to be duplicated to form small families is not clear.

The divergent topologies of the trees for the NACHT and B30.2 domains (electronic supplementary material, figure S7) suggest different evolutionary paths for the domains, and these are confirmed by analysis of substitution rates. We see low rates of synonymous substitutions in the NACHT domain when comparing the members within each group, and similarly low rates of synonymous substitutions for the B30.2 domains in comparisons across all groups. A low rate of synonymous sequence substitutions can be interpreted as a sign of recent gene duplication. If we apply this interpretation using the low divergence of the B30.2 domain, then we would have to conclude that the entire set of genes in groups 1, 2a and 3a is the product of recent duplications. However, this is not consistent with the significant divergence of the NACHT domains between the groups, the different tree topologies of the two domains, or with our finding that the split into NLR families occurred before the divergence of zebrafish and carp. Therefore, there must be an alternative explanation. The pattern of synonymous divergence of the two domains between groups is most parsimoniously explained by ongoing gene conversion.

Gene conversion in the NACHT domain appears to be restricted to conversion within each group, keeping the groups homogeneous and distinct from each other. In contrast, gene conversion between B30.2 domains may have another effect, namely to create additional variation. The process is not uncommon in gene families involved in immunity (see [30] for review). It can create diversity, for example, in antibodies [31] or in the MHC (reviewed in [32]), but it can also homogenize genes, e.g. in the T-cell receptor family [33]. In the NLR-B30.2 family, both mechanisms may operate.

The high dN/dS values indicate positive selection for non-synonymous variants in residues potentially involved in pathogen recognition. The substitutions are concentrated in the same hypervariable regions of the B30.2 domain in which the variation is also seen in the fintrims [14]. These correspond to regions exposed on the surface of TRIM5 [34] and are therefore presumed to be involved in pathogen interactions. Once substitutions have been introduced in one of the genes, gene conversion can then spread these throughout the family. If conversion tracts are shorter than the entire B30.2 exon, substitutions occurring in different parts can be combined, creating additional variation. At the same time, because gene conversion in the B30.2 domain acts across groups, this mechanism also ensures that new recognition modules can spread beyond the group in which they first arose. This can prevent the groups from being characterized by a defined subset of B30.2 domains. It is striking that the three groups of genes that show gene conversion in the B30.2 domain are all localized on chromosome 4, whereas group 3b, which has diverged from group 3a in its B30.2 domain, is located on chromosome 1.

At this point, gene conversion may already have been occurring and if the early prototypes had already amplified into gene families in the common ancestor, then gene conversion may have acted within each group. Gene conversion must have stopped occurring between NACHT domains of different groups to allow for the observed divergence, but continued in the B30.2 domains. Because not all currently extant fish have representatives of all four groups, it may be that either whole sets of these genes can be easily lost, or else that the common precursor had only one gene from each group, and that not all lineages inherited all four prototypes. The near-identity of some of the genes we find in zebrafish (difference between paralogues lower than the rate of polymorphism) shows that duplications continue to occur.

It is worth speculating about the functional and selective forces that prevent sequence homogenization between the NACHT domains of different groups. If the proteins form large multimeric complexes, as the known inflammasome NLRs do, then their efficient functioning might require that only proteins from the same group can multimerize, for instance to elicit distinct downstream signalling events. This is supported by the fact that the group members feature different N-terminal domains. A mixed multimer may not be able to assemble a functional N-terminal effector complex.

The C-terminal domains—LRRs and B30.2—do not show the same clear subdivision into families as the N-terminal and the NACHT domains, and homogenizing gene conversion must therefore have affected only part of each gene, or affected separate parts differently. This is not without precedent, because gene conversion often proceeds across DNA segments of limited length (see [35] for review) and parts of a gene can escape sequence homogenization [35,36].

Both LRRs and B30.2 domains have been implicated in recognition of pathogen- or danger-associated molecular patterns. The B30.2 domain of TRIM5a binds to HIV-1 and is involved in blocking HIV-1 proliferation in monkeys [37]. LRRs have been implicated in the recognition of pathogen-associated molecular patterns both in the LRR-containing transmembrane proteins of the Toll-like receptor proteins, and in the NLR proteins in plants and animals (reviewed in [38–40]).

The sequences of the LRRs in the NLR-B30.2 genes are not particularly variable, and it therefore seems unlikely that they have a role in specific ligand recognition. The B30.2 domains, however, show significant amino acid variation between the members. It may therefore have the same function as the related B30.2 domain in the *fintrim* genes, which has been suggested to be under positive selection to allow variation in specificity for pathogen recognition [11]. It is conceivable that the acquisition of the B30.2 domain and the option to use it for specific recognition of a wide range of pathogens drove the amplification of these genes.

Not many salt-water fish genome assemblies are available. We did not find the NLR-B30.2 genes in the Atlantic cod, but the Atlantic salmon (which spends a good part of its life cycle in fresh water) has a set of approximately 20 representatives. We are tempted to speculate that the massive inflation of the NLR-B30.2 group may be associated with the adaptation to fresh water environments. Alternatively, the NLR-B30.2 system may functionally complement the adaptive immune system during the first few weeks of life of the zebrafish larva: the larva is exposed to the outside world and starts eating after two days of development, but a functional adaptive immune systems arises only after three to five weeks [41]. We have not investigated whether the presence of NLR-B30.2 expansions in a fish species correlates with the time of development of the adaptive immune system in that species.

### 3.2. Shuffling between genes and creation of new genes

A mechanism involved in the initial creation of the NLR-B30.2 family appears to have been exon shuffling, both within the family and between the NLR genes and other gene families. For example, the N-terminal peptide repeats occur in several variants, but a given variant is not strictly associated with any particular group: at least two of the variants are found in association with both group 2a and group 3.

We also find evidence for recombination with other immune genes. The B30.2 domain of the NLR-B30.2 proteins most closely resembles that of the fintrim proteins, a fish-specific gene family for which the origin of the fusion between the Zn-fingers with the B30.2 domain is not known [14]. This suggests that exon shuffling occurred during the generation of the ancestral genes of the NLR-B30.2 and the *fintrim* gene families.

Apart from this possible case of exon exchange, the relationship between the three large and partially related families—the NLR-B30.2 genes, the *fintrim* genes and the multi-Zn-finger genes we describe here—are unclear. While it is striking that the fintrims share the B30.2 domain with the NLR-B30.2 genes and the Zn-fingers with the multi-Zn-finger genes, they do not preferentially map to the same regions of the genome, and the Zn-finger is of a different type. By contrast, the multi-Zn-finger genes are mostly found on chromosome 4, interspersed between the NLR-B30.2 genes.

A further gene that may have arisen from domain shuffling between these gene families is the human gene encoding pyrin (marenostrin/MEFV). Pyrin is a protein that is composed of an N-terminal PYD domain, for which the best match in the zebrafish is the PYD domain in the group 1 NLR proteins. The C-terminal part of pyrin contains a Zn-finger and a B30.2 domain, which resembles the zebrafish fintrim proteins of the btr family. The most likely interpretation for the origin of this gene, which must have arisen at the base of the tetrapods, is therefore a recombination between an NLR gene and a neighbouring *fintrim* gene.

### 3.3. Chromosome 4

The zebrafish chromosome 4 has unusual properties. Its long arm is entirely heterochromatic, replicates late and shows a reduced recombination rate. It contains an accumulation of 5S rRNA, snRNA, tRNA and mir-430 clusters [42,43], as well as the expanded protein coding gene families described here.

Chromosome 4 was recently shown to function as the sex chromosome in wild zebrafish ZW/WW sex determination, with the sex determining signal being located towards the telomere of the long arm of chromosome 4 [44]. The sex determination region in the grass carp may also be associated with NACHT domain encoding genes [45]. This was concluded from the comparison of the genome sequences of one male and one female carp, where those regions present in the male and absent in the female were interpreted as sex determining. In addition to the NACHT domain genes, this region also included other immunity genes, such as the immunoglobulin V-set, ABC transporters and proteasome subunits. While the co-location between sex determination and immune signalling molecules we describe here may support this conclusion, it is of course equally possible that the finding in the grass carp is simply caused by allelic diversity in these highly variable genes between the two individuals. It is nevertheless intriguing that two fast evolving genetic systems are located in such close proximity in zebrafish. Perhaps, after an initial round of NLR gene duplications, a run-away evolutionary process of further amplification created the present chromosome 4, which is now a hotspot for rapid evolutionary processes.

## 4. Methods

### 4.1. Re-annotation of NLR genes in the zebrafish genome

To establish a complete list of all genes encoding NLR proteins in the zebrafish genome, we first conducted a search of the Zv9 genome assembly for sequences that encoded the characteristic protein domains, using a combination of approaches. We constructed a hidden Markov model (HMM) for the Fisna domain and used this together with the HMM for the NACHT domain obtained from PFAM to search the Zv9 assembly with hmmsearch (hmmer.janelia.org/search/hmmsearch), resulting in 297 Fisna and 328 NACHT locations (see the electronic supplementary material). As an alternative approach to identify NACHT domains specific for the novel NLRs, we ran electronic PCRs [15] with primer sets for a segment stretching from the C-domain into the winged helix domain that we had used for experimental analysis of the genes (2010, unpublished work). Each set of primers was specific for one of the NLR groups (electronic supplementary material, Methods). This resulted in 321 hits. To find regions in the genome encoding B30.2 domains, we conducted a TBLASTN search, which yielded 503 hits. As B30.2 domains also occur in other large, immune-related protein families (see below), such a high number of domains was consistent with expectations.

Second, we collected all Ensembl genes overlapping the above motifs (487 predicted genes) and also all manually annotated genes (vega.sanger.ac.uk) that had been marked as NLR or as containing a NACHT domain during manual annotations in the past (307 predicted genes).

The collection was purged of gene models that did not match the criteria for being novel NLRs, excluding e.g. the B30.2 domain-containing *fintrim* genes. Sixteen NACHT domain proteins in the combined list do not belong to the group of novel fish NLRs because they do not contain the Fisna domain, and the sequence surrounding their Walker A motifs does not match the one typical for the novel NLRs. They include the eight conserved NLRs that are orthologous across all vertebrate species (Nod1, Nod2, Nlrc3, Nlrx1, CIITA, Apaf1, NWD1/NachtP1), and nine further proteins with an NLR structure (table 1 and electronic supplementary material).

Comparison of the purged gene sets with the genomic regions that encoded parts of NLR proteins showed that many genes in this family had been annotated incorrectly, and for others there were no predictions at all, probably owing to the repetitive nature of this gene family and the limited availability of supporting evidence in the form of cDNAs.

The regions containing the sequences identified in our searches were therefore re-annotated manually as described elsewhere [46,47], correcting and adding gene models to create full-length genes. This re-annotation had to be restricted to regions located on finished sequence, because whole genome shotgun contigs in Zv9 were not accessible to manual annotation. For these contigs, the automated Ensembl gene models were retained in their original form, recognizable in our final list by their ‘ENSDARG’ identifier (electronic supplementary material, table S1). The resulting protein sequences were then aligned using Clustal–Omega [48] or Muscle [49] and compared. Sequences that appeared truncated were analysed further by searching for additional exons to complete them, until, in an iterative process, we had optimized them. Some sequences remained incomplete, either because they were located next to sequence gaps, or because no additional exons could be detected. In these cases, it is not known whether the truncation of the gene is a true biological event caused by recent recombination, or whether it is due to a misassembly of the genome sequence.

The optimized gene set was combined with the gene predictions in Ensembl (Methods, hand-filtered alignments and location checks of the remaining genes to identify accordance). The final list of novel zebrafish NLR proteins contains 368 members (electronic supplementary material, table S1). A further 36 predictions for NLR genes had been annotated as pseudo-genes and were therefore not retrieved for this list (electronic supplementary material). The refined genes have since been integrated into the VEGA and Ensembl gene sets. However, because the annotation was performed on pre-GRCz10 paths, the latest GRCz10 gene set (Ensembl80) might differ marginally from the described results.

### 4.2. Conserved NLR genes across metazoa

We used the zebrafish gene identifiers for the conserved NLRs in zebrafish to query the Orthoinspector v. 2.0 database [50] at http://lbgi.igbmc.fr/orthoinspector for orthologues in published genomes and downloaded the corresponding sequence. We then queried a custom Blast database of the *Cyprinus carpio* proteome, as well as the NCBI nr database for selected fish species using BLASTP. After removing redundant hits, we calculated alignments employing Clustal–Omega v. 1.2 [48] and subsequently removed sequences of poor quality. In a second inference, we also used trimal [51] to reduce the alignment to the conserved residues. We employed prottest v. 3.2 [52] to infer the best fitting evolutionary model and found that the LG model with Gamma optimization performed best under the Akaike information criterion. We then ran RAxML v. 7.7.2 [53] on both alignments on the Cologne University CHEOPS super computer and calculated bootstrap values. Phylogenetic trees were visualized and edited in Dendroscope v. 3.2.5 [54].

### 4.3. Figmop and TBLASTN screen for NLR-B30.2 candidates in other fish genomes

Expanded gene families are not well annotated in most genomes. Rather than relying on gene predictions for identifying NLR-B30.2 genes, we therefore directly searched the genome sequences of six species: *Latimeria chalumnae, Lepisosteus oculatus, Callorhinchus milii, Esox lucius, Astyanax mexicanus* and *Cyprinus carpio*. We downloaded genome data either from NCBI servers or the genome project websites. We then used the Figmop [55] pipeline to find contigs and scaffolds in the genomes with NLR-B30.2 candidates on them. The Figmop pipeline builds a profile of conserved motifs from a starting set of sequences and uses these to search a target database with the MEME software suite [56].

We used zebrafish NLR-B30.2 sequences from all four groups to create a set of 15 motifs to search the above genomes. The resulting contigs were then subjected to the Augustus (v. 3.0.3) gene prediction pipeline [57] to predict genes de novo, setting zebrafish as the ‘species’. We complemented this approach by TBLASTN searches using the NACHT as well as the B30.2 domains as queries in individual searches and then kept those predictions in which the domains occurred in the proper order (thereby excluding spurious cases caused by misassembly or incomplete genes).

### 4.4. Phylogenetic analyses of the NLR-B30.2 groups

We used a recursive approach for identifying genes for the phylogeny that were representative of the overall sequence divergence in the gene family. We selected only those that had both the NACHT and B30.2 domains. We then recursively performed the following: (i) constructed a sequence alignment of approximately 500 residues (starting with the NACHT domain) in the dataset using Clustal–Omega, (ii) constructed a phylogeny using a Bayesian approach using MrBayes with mcmc = 1 000 000, sump burnin = 1000 and sumt burnin = 1000 and (iii) removed monophyletic paralogues from the dataset. The recursive analysis was halted when no instances of paralogous sister sequences remained (figure 3), with the exception that at least one zebrafish and one carp sequence from each of the major groups was retained. Once the final dataset of sequences was determined we removed gap-containing and highly variable columns from the alignment and re-ran MrBayes with mcmc = 2 000 000, sump burnin = 2000 and sumt burnin = 2000 and re-confirmed our inferred tree with maximum-likelihood in RAxML.

We also used RAxML to infer a phylogeny of all currently available *D. rerio* and *C. carpio* NLR-B30.2 genes. As described for the conserved NLR genes above we based our phylogeny on an alignment calculated with Clustal–Omega v. 1.2, reduced to conserved regions with trimal, and model testing with prottest (JTT + G + F model found to be optimal).

### 4.5. Divergence analysis

For zebrafish genes in groups 1, 2a, 3a and 3b, we calculated all pairwise dN/dS values for NACHT domain containing exons and the B30.2 domain independently using the Ka/Ks calculator [58]: we extracted the respective regions from our protein alignment, then used tranalign [59] to create DNA alignments from these proteins and cds. We then calculated all pairwise comparisons and used paraAT [60] and submitted the resulting alignments to the Ka/Ks calculator independently estimating under the MYN model and the model averaging option with aid of the gnu-parallel tool [61]. We then used our own iPython [62] script to sort data and calculated means, medians and errors in the R statistic software (R Core Team 2015).

We also used the tranaligned regions to calculate independent phylogenies for the NACHT and B30.2 exons with RAxML. We loaded the inferred trees into Dendroscope and employed this software to visualize connection between branches belonging to the same gene in both trees.

## Data accessibility

All online supplementary material can be found on Figshare under the accession number http://dx.doi.org/10.6084/m9.figshare.1473092.

## References

[1] Koonin EV, Aravind L 2000 The NACHT family – a new group of predicted NTPases implicated in apoptosis and MHC transcription activation. Trends Biochem. Sci. 25, 223–224. (doi:10.1016/S0968-0004(00)01577-2) 10782090 10.1016/s0968-0004(00)01577-2

[2] Proell M, Riedl SJ, Fritz JH, Rojas AM, Schwarzenbacher R 2008 The Nod-like receptor (nlr) family: a tale of similarities and differences. PLoS ONE 3, e2119 (doi:10.1371/journal.pone.0002119) 18446235 10.1371/journal.pone.0002119 PMC2323615

[3] Ting JPY et al. 2008 The NLR gene family: a standard nomenclature. Immunity 28, 285–287. (doi:10.1016/j.immuni.2008.02.005) 18341998 10.1016/j.immuni.2008.02.005 PMC2630772

[4] Fritz JH, Girardin SE 2005 How Toll-like receptors and Nod-like receptors contribute to innate immunity in mammals. J. Endotoxin Res. 11, 390–394. (doi:10.1177/09680519050110060301) 16303096 10.1179/096805105X76850

[5] Creagh EM, O'Neill LAJ 2006 TLRs, NLRs and RLRs: a trinity of pathogen sensors that co-operate in innate immunity. Trends Immunol. 27, 352–357. (doi:10.1016/j.it.2006.06.003) 16807108 10.1016/j.it.2006.06.003

[6] Netea MG, Azam T, Ferwerda G, Girardin SE, Kim S-H, Dinarello CA 2006 Triggering receptor expressed on myeloid cells-1 (TREM-1) amplifies the signals induced by the NACHT-LRR (NLR) pattern recognition receptors. J. Leukoc. Biol. 80, 1454–1461. (doi:10.1189/jlb.1205758) 16940328 10.1189/jlb.1205758

[7] Stein C, Caccamo M, Laird G, Leptin M 2007 Conservation and divergence of gene families encoding components of innate immune response systems in zebrafish. Genome Biol. 8, R251 (doi:10.1186/gb-2007-8-11-r251) 18039395 10.1186/gb-2007-8-11-r251 PMC2258186

[8] Kufer TA, Sansonetti PJ 2011 NLR functions beyond pathogen recognition. Nat. Immunol. 12, 121–128. (doi:10.1038/ni.1985) 21245903 10.1038/ni.1985

[9] Bonardi V, Cherkis K, Nishimura MT, Dangl JL 2012 A new eye on NLR proteins: focused on clarity or diffused by complexity? Curr. Opin. Immunol. 24, 41–50. (doi:10.1016/j.coi.2011.12.006) 22305607 10.1016/j.coi.2011.12.006 PMC3482489

[10] Liu Y, Zhang Y-B, Liu T-K, Gui J-F 2013 Lineage-specific expansion of IFIT gene family: an insight into coevolution with IFN gene family. PLoS ONE 8, e66859 (doi:10.1371/journal.pone.0066859) 23818968 10.1371/journal.pone.0066859 PMC3688568

[11] Rowe HM, Withey JH, Neely MN 2014 Zebrafish as a model for zoonotic aquatic pathogens. Dev. Comp. Immunol. 46, 96–107. (doi:10.1016/j.dci.2014.02.014) 24607289 10.1016/j.dci.2014.02.014 PMC4096445

[12] Goody MF, Sullivan C, Kim CH 2014 Studying the immune response to human viral infections using zebrafish. Dev. Comp. Immunol. 46, 84–95. (doi:10.1016/j.dci.2014.03.025) 24718256 10.1016/j.dci.2014.03.025 PMC4067600

[13] Laing KJ, Purcell MK, Winton JR, Hansen JD 2008 A genomic view of the NOD-like receptor family in teleost fish: identification of a novel NLR subfamily in zebrafish. BMC Evol. Biol. 8, 42 (doi:10.1186/1471-2148-8-42) 18254971 10.1186/1471-2148-8-42 PMC2268669

[14] van der Aa LM, Levraud J-P, Yahmi M, Lauret E, Briolat V, Herbomel P, Benmansour A, Boudinot P 2009 A large new subset of TRIM genes highly diversified by duplication and positive selection in teleost fish. BMC Biol. 7, 7 (doi:10.1186/1741-7007-7-7) 19196451 10.1186/1741-7007-7-7 PMC2657112

[15] Schuler GD 1997 Sequence mapping by electronic PCR. Genome Res. 7, 541–550. 9149949 10.1101/gr.7.5.541 PMC310656

[16] Rast JP, Buckley KM 2013 Lamprey immunity is far from primitive. Proc. Natl Acad. Sci. USA 110, 5746–5747. (doi:10.1073/pnas.1303541110) 23553834 10.1073/pnas.1303541110 PMC3625256

[17] Yuen B, Bayes JM, Degnan SM 2014 The characterization of sponge NLRs provides insight into the origin and evolution of this innate immune gene family in animals. Mol. Biol. Evol. 31, 106–120. (doi:10.1093/molbev/mst174) 24092772 10.1093/molbev/mst174 PMC3879445

[18] Lapraz F et al. 2006 RTK and TGF-β signaling pathways genes in the sea urchin genome. Dev. Biol. 300, 132–152. (doi:10.1016/j.ydbio.2006.08.048) 17084834 10.1016/j.ydbio.2006.08.048 PMC12337106

[19] Howe K, Wood JM 2015 Using optical mapping data for the improvement of vertebrate genome assemblies. Gigascience 4, 10 (doi:10.1186/s13742-015-0052-y) 25789164 10.1186/s13742-015-0052-y PMC4364110

[20] Sola L, Gornung E 2001 Classical and molecular cytogenetics of the zebrafish, *Danio rerio* (Cyprinidae, Cypriniformes): an overview. Genetica 111, 397–412. (doi:10.1023/A:1013776323077) 11841183 10.1023/a:1013776323077

[21] van der Aa LM, Jouneau L, Laplantine E, Bouchez O, Van Kemenade L, Boudinot P 2012 FinTRIMs, fish virus-inducible proteins with E3 ubiquitin ligase activity. Dev. Comp. Immunol. 36, 433–441. (doi:10.1016/j.dci.2011.08.010) 21907235 10.1016/j.dci.2011.08.010

[22] Correa RG, Krajewska M, Ware CF, Gerlic M, Reed JC 2014 The NLR-related protein NWD1 is associated with prostate cancer and modulates androgen receptor signaling. Oncotarget 5, 1666–1682. (doi:10.18632/oncotarget.1850) 24681825 10.18632/oncotarget.1850 PMC4039239

[23] Huang S et al. 2008 Genomic analysis of the immune gene repertoire of amphioxus reveals extraordinary innate complexity and diversity. Genome Res. 18, 1112–1126. (doi:10.1101/gr.069674.107) 18562681 10.1101/gr.069674.107 PMC2493400

[24] Kondrashov FA, Rogozin IB, Wolf YI, Koonin EV 2002 Selection in the evolution of gene duplications. Genome Biol. 3, research0008.1. (doi:10.1186/gb-2002-3-2-research0008) 10.1186/gb-2002-3-2-research0008 PMC65685 11864370

[25] Kondrashov FA 2012 Gene duplication as a mechanism of genomic adaptation to a changing environment. Proc. R. Soc. B 279, 5048–5057. (doi:10.1098/rspb.2012.1108) 10.1098/rspb.2012.1108 PMC3497230 22977152

[26] Chen Z et al. 2008 Transcriptomic and genomic evolution under constant cold in Antarctic notothenioid fish. Proc. Natl Acad. Sci. USA 105, 12 944–12 949. (doi:10.1073/pnas.0802432105) 10.1073/pnas.0802432105 PMC2529033 18753634

[27] Li J, Das S, Herrin BR, Hirano M, Cooper MD 2013 Definition of a third VLR gene in hagfish. Proc. Natl Acad. Sci. USA 110, 15 013–15 018. (doi:10.1073/pnas.1314540110) 10.1073/pnas.1314540110 PMC3773805 23980174

[28] Hamada M, Shoguchi E, Shinzato C, Kawashima T, Miller DJ, Satoh N 2012 The complex NOD-like receptor repertoire of the coral *Acropora digitifera* includes novel domain combinations. Mol. Biol. Evol. 30, 167–176. (doi:10.1093/molbev/mss213) 10.1093/molbev/mss213 22936719

[29] Sardiello M, Cairo S, Fontanella B, Ballabio A, Meroni G 2008 Genomic analysis of the TRIM family reveals two groups of genes with distinct evolutionary properties. BMC Evol. Biol. 8, 225 (doi:10.1186/1471-2148-8-225) 18673550 10.1186/1471-2148-8-225 PMC2533329

[30] Pasquier LD 2006 Germline and somatic diversification of immune recognition elements in metazoa. Immunol. Lett. 104, 2–17. (doi:10.1016/j.imlet.2005.11.022) 16388857 10.1016/j.imlet.2005.11.022

[31] Wysocki LJ, Gefter ML 1989 Gene conversion and the generation of antibody diversity. Annu. Rev. Biochem. 58, 509–531. (doi:10.1146/annurev.bi.58.070189.002453) 2673016 10.1146/annurev.bi.58.070189.002453

[32] Martinsohn JT, Sousa AB, Guethlein LA, Howard JC 1999 The gene conversion hypothesis of MHC evolution: a review. Immunogenetics 50, 168–200. (doi:10.1007/s002510050593) 10602879 10.1007/s002510050593

[33] Jouvin-Marche E, Heller M, Rudikoff S 1986 Gene correction in the evolution of the T cell receptor beta chain. J. Exp. Med. 164, 2083–2088. (doi:10.1084/jem.164.6.2083) 3783089 10.1084/jem.164.6.2083 PMC2188480

[34] Woo J-S, Suh H-Y, Park S-Y, Oh B-H 2006 Structural basis for protein recognition by B30.2/SPRY domains. Mol. Cell 24, 967–976. (doi:10.1016/j.molcel.2006.11.009) 17189197 10.1016/j.molcel.2006.11.009

[35] Innan H 2009 Population genetic models of duplicated genes. Genetica 137, 19–37. (doi:10.1007/s10709-009-9355-1) 19266289 10.1007/s10709-009-9355-1

[36] Teshima KM, Innan H 2004 The effect of gene conversion on the divergence between duplicated genes. Genetics 166, 1553–1560. (doi:10.1534/genetics.166.3.1553) 15082568 10.1534/genetics.166.3.1553 PMC1470786

[37] Stremlau M, Owens CM, Perron MJ, Kiessling M, Autissier P, Sodroski J 2004 The cytoplasmic body component TRIM5α restricts HIV-1 infection in old world monkeys. Nature 427, 848–853. (doi:10.1038/nature02343) 14985764 10.1038/nature02343

[38] Inohara N, Chamaillard M, McDonald C, Nunez G 2005 NOD-LRR proteins: role in host–microbial interactions and inflammatory disease. Annu. Rev. Biochem. 74, 355–383. (doi:10.1146/annurev.biochem.74.082803.133347) 15952891 10.1146/annurev.biochem.74.082803.133347

[39] Akira S, Uematsu S, Takeuchi O 2006 Pathogen recognition and innate immunity. Cell 124, 783–801. (doi:10.1016/j.cell.2006.02.015) 16497588 10.1016/j.cell.2006.02.015

[40] Kawai T, Akira S 2009 The roles of TLRs, RLRs and NLRs in pathogen recognition. Int. Immunol. 21, 317–337. (doi:10.1093/intimm/dxp017) 19246554 10.1093/intimm/dxp017 PMC2721684

[41] Lam SH, Chua HL, Gong Z, Lam TJ, Sin YM 2004 Development and maturation of the immune system in zebrafish, *Danio rerio*: a gene expression profiling, in situ hybridization and immunological study. Dev. Comp. Immunol. 28, 9–28. (doi:10.1016/S0145-305X(03)00103-4) 12962979 10.1016/s0145-305x(03)00103-4

[42] Anderson JL, Marí AR, Braasch I, Amores A, Hohenlohe P, Batzel P, Postlethwait JH 2012 Multiple sex-associated regions and a putative sex chromosome in zebrafish revealed by RAD mapping and population genomics. PLoS ONE 7, e40701 (doi:10.1371/journal.pone.0040701) 22792396 10.1371/journal.pone.0040701 PMC3392230

[43] Howe K et al. 2013 The zebrafish reference genome sequence and its relationship to the human genome. Nature 496, 498–503. (doi:10.1038/nature12111) 23594743 10.1038/nature12111 PMC3703927

[44] Wilson CA et al. 2014 Wild sex in zebrafish: loss of the natural sex determinant in domesticated strains. Genetics 198, 1291–1308. (doi:10.1534/genetics.114.169284) 25233988 10.1534/genetics.114.169284 PMC4224167

[45] Wang Y et al. 2015 The draft genome of the grass carp (*Ctenopharyngodon idellus*) provides insights into its evolution and vegetarian adaptation. Nat. Genet. 47, 625–631. (doi:10.1038/ng.3280) 25938946 10.1038/ng.3280

[46] Mudge JM, Harrow J 2015 Creating reference gene annotation for the mouse C57BL6/J genome assembly. Mamm. Genome 26, 366–378. (doi:10.1007/s00335-015-9583-x) 26187010 10.1007/s00335-015-9583-x PMC4602055

[47] Jekosch K 2004 The zebrafish genome project: sequence analysis and annotation. In The zebrafish: genetics, genomics and informatics. Methods in Cell Biology, vol. 77 (eds HW Detrich, M Westerfield, LI Zon), pp. 225–239. San Diego, CA: Elsevier Academic Press. 10.1016/s0091-679x(04)77012-0 15602914

[48] Sievers F et al. 2011 Fast, scalable generation of high-quality protein multiple sequence alignments using Clustal Omega. Mol. Syst. Biol. 7, 1–6. (doi:10.1038/msb.2011.75) 10.1038/msb.2011.75 PMC3261699 21988835

[49] Edgar RC 2004 MUSCLE: a multiple sequence alignment method with reduced time and space complexity. BMC Bioinformatics 5, 113 (doi:10.1186/1471-2105-5-113) 15318951 10.1186/1471-2105-5-113 PMC517706

[50] Linard B, Allot A, Schneider R, Morel C, Ripp R, Bigler M, Thompson JD, Poch O, Lecompte O 2014 OrthoInspector 2.0: software and database updates. Bioinformatics 31, 447–448. (doi:10.1093/bioinformatics/btu642) 25273105 10.1093/bioinformatics/btu642

[51] Capella-Gutiérrez S, Silla-Martínez JM, Gabaldón T 2009 trimAl: a tool for automated alignment trimming in large-scale phylogenetic analyses. Bioinformatics 25, 1972–1973. (doi:10.1093/bioinformatics/btp348) 19505945 10.1093/bioinformatics/btp348 PMC2712344

[52] Darriba D, Taboada GL, Doallo R, Posada D 2011 ProtTest 3: fast selection of best-fit models of protein evolution. Bioinformatics 27, 1164–1165. (doi:10.1093/bioinformatics/btr088) 21335321 10.1093/bioinformatics/btr088 PMC5215816

[53] Stamatakis A 2006 RAxML-VI-HPC: maximum likelihood-based phylogenetic analyses with thousands of taxa and mixed models. Bioinformatics 22, 2688–2690. (doi:10.1093/bioinformatics/btl446) 16928733 10.1093/bioinformatics/btl446

[54] Huson DH, Richter DC, Rausch C, Dezulian T, Franz M, Rupp R 2007 Dendroscope: an interactive viewer for large phylogenetic trees. BMC Bioinformatics 8, 460 (doi:10.1186/1471-2105-8-460) 18034891 10.1186/1471-2105-8-460 PMC2216043

[55] Curran DM, Gilleard JS, Wasmuth JD 2014 Figmop: a profile HMM to identify genes and bypass troublesome gene models in draft genomes. Bioinformatics 30, 3266–3267. (doi:10.1093/bioinformatics/btu544) 25115706 10.1093/bioinformatics/btu544

[56] Bailey TL, Boden M, Buske FA, Frith M, Grant CE, Clementi L, Ren J, Li WW, Noble WS 2009 MEME Suite: tools for motif discovery and searching. Nucleic Acids Res. 37, W202–W208. (doi:10.1093/nar/gkp335) 19458158 10.1093/nar/gkp335 PMC2703892

[57] Stanke M, Waack S 2003 Gene prediction with a hidden Markov model and a new intron submodel. Bioinformatics 19, ii215–ii225. (doi:10.1093/bioinformatics/btg1080) 14534192 10.1093/bioinformatics/btg1080

[58] Wang D, Zhang Y, Zhang Z, Zhu J, Yu J 2010 KaKs_calculator 2.0: a toolkit incorporating gamma-series methods and sliding window strategies. Genomics Proteomics Bioinformatics 8, 77–80. (doi:10.1016/S1672-0229(10)60008-3) 20451164 10.1016/S1672-0229(10)60008-3 PMC5054116

[59] Rice P, Longden I, Bleasby A 2000 EMBOSS: the European molecular biology open software suite. Trends Genet. 16, 276–277. (doi:10.1016/S0168-9525(00)02024-2) 10827456 10.1016/s0168-9525(00)02024-2

[60] Zhang Z, Xiao J, Wu J, Zhang H, Liu G, Wang X, Dai L 2012 ParaAT: a parallel tool for constructing multiple protein-coding DNA alignments. Biochem. Biophys. Res. Commun. 419, 779–781. (doi:10.1016/j.bbrc.2012.02.101) 22390928 10.1016/j.bbrc.2012.02.101

[61] Tange O 2011 Gnu parallel: the command-line power tool. The USENIX Magazine. See https://www.usenix.org/publications/login/february-2011-volume-36-number-1/gnu-parallel-command-line-power-tool.

[62] Pérez F, Granger BE 2007 IPython: a system for interactive scientific computing. Comput. Sci. Eng. 9, 21–29. (doi:10.1109/MCSE.2007.53)

